# Attachment Patterns in Children and Adolescents With Gender Dysphoria

**DOI:** 10.3389/fpsyg.2020.582688

**Published:** 2021-01-12

**Authors:** Kasia Kozlowska, Catherine Chudleigh, Georgia McClure, Ann M. Maguire, Geoffrey R. Ambler

**Affiliations:** ^1^Department of Psychological Medicine, The Children’s Hospital at Westmead, Westmead, NSW, Australia; ^2^Discipline of Child and Adolescent Health, University of Sydney Medical School, Darlington, NSW, Australia; ^3^Department of Endocrinology, The Children’s Hospital at Westmead, Westmead, NSW, Australia

**Keywords:** attachment, gender dysphoria, transgender, dynamic maturation model of attachment (DMM), children and adolescents

## Abstract

The current study examines patterns of attachment/self-protective strategies and rates of unresolved loss/trauma in children and adolescents presenting to a multidisciplinary gender service. Fifty-seven children and adolescents (8.42–15.92 years; 24 birth-assigned males and 33 birth-assigned females) presenting with gender dysphoria participated in structured attachment interviews coded using dynamic-maturational model (DMM) discourse analysis. The children with gender dysphoria were compared to age- and sex-matched children from the community (non-clinical group) and a group of school-age children with mixed psychiatric disorders (mixed psychiatric group). Information about adverse childhood experiences (ACEs), mental health diagnoses, and global level of functioning was also collected. In contrast to children in the non-clinical group, who were classified primarily into the normative attachment patterns (A1-2, B1-5, and C1-2) and who had low rates of unresolved loss/trauma, children with gender dysphoria were mostly classified into the high-risk attachment patterns (A3-4, A5-6, C3-4, C5-6, and A/C) (χ^2^ = 52.66; *p* < 0.001) and had a high rate of unresolved loss/trauma (χ^2^ = 18.64; *p* < 0.001). Comorbid psychiatric diagnoses (*n* = 50; 87.7%) and a history of self-harm, suicidal ideation, or symptoms of distress were also common. Global level of functioning was impaired (range 25–95/100; mean = 54.88; *SD* = 15.40; median = 55.00). There were no differences between children with gender dysphoria and children with mixed psychiatric disorders on attachment patterns (χ^2^ = 2.43; *p* = 0.30) and rates of unresolved loss and trauma (χ^2^ = 0.70; *p* = 0.40). *Post hoc* analyses showed that lower SES, family constellation (a non-traditional family unit), ACEs—including maltreatment (physical abuse, sexual abuse, emotional abuse, neglect, and exposure to domestic violence)—increased the likelihood of the child being classified into a high risk attachment pattern. Akin to children with other forms of psychological distress, children with gender dysphoria present in the context of multiple interacting risk factors that include at-risk attachment, unresolved loss/trauma, family conflict and loss of family cohesion, and exposure to multiple ACEs.

## Introduction

Over the last few decades, in Western clinical practice, children (including adolescents) with gender dysphoria have emerged as a poorly understood clinical population with increasing presentations to health services (Zucker, 2017; Meyer-Bahlburg, 2019). Children with gender dysphoria experience significant distress because their gender identity—their subjective experience of the self as male or female (or other)—does not correspond to their sex assigned at birth. Clinicians working from a broad biopsychosocial, or systems, perspective (Engel, 1980; The Forum for Youth Investment, 2020; Capra and Luisi, 2014) are interested in understanding the processes—biological, psychological, relational, and cultural—that have come together to shape the child’s developmental pathway. In this article we examine one thread of the developmental story—the quality of the child’s attachment relationships within the family system—in a cohort of children with dysphoria and subjective distress about their birth-assigned sex who have presented for assessment and treatment to a multidisciplinary service in a tertiary care hospital.

John Bowlby, the father of attachment theory, used the analogy of branching train tracks in a railway yard to think about developmental pathways and patterns of adaptation and maladaptation (Bowlby, 1973a). He conceptualized the problems of troubled children as emerging from a complex interplay between genetic factors, experience—the intrauterine experience, the quality of the child’s attachment relationships, the family experience, and the impact of adverse childhood experiences (ACEs)—and sociopolitical and cultural factors. Bowlby emphasized that developmental pathways did not follow a linear pattern (see Box 1) (Bowlby, 1973a, 1988): the child’s developmental pathway “turns at each and every stage of the journey on an interaction between the organism as it has developed up to that moment and the environment in which it then finds itself” (p 419) (Bowlby, 1973b). His work set the foundations for developmental models of brain development and psychopathology (Perry, 2009; Masten and Cicchetti, 2010; Sroufe, 2013). These models conceptualize development—and brain development, in particular—as cumulative; it builds upon itself, albeit in a non-linear way (Stiles, 2008). Each developmental phase, coupled with each child’s lived experiences, provides a foundation for the next; past development shapes subsequent development. Subsequently, prospective, population-based studies have shown that poor-quality attachment relationships (at-risk patterns of attachment) are a risk factor for psychopathology later in life (Sroufe, 2005).

**Box1. The characteristics of developmental pathways as outlined by Bowlby.**

• Any starting pathway can have numerous possible outcomes (multifinality).

• Two different starting pathways can lead to the same outcome (equifinality).

• Change in pathway remains possible across development.

• Change is constrained by how long a pathway had been followed.

• Maladaptive development is defined by sustained deviation from functional pathways.

More recently, and more broadly, neuroscience research has begun to elucidate some of the neurobiological mechanisms by which lived experiences, including those relating to attachment, shape the developing brain, the expression of genes, and the array of capacities that allow the child to cope effectively or ineffectively with the developmental challenges of each developmental period (Stiles, 2008; Dudley et al., 2011; Babenko et al., 2015; Shonkoff, 2016). Because early experiences alter brain structure and function as well as genetic expression, they become *biologically embedded* or *internalized* into the child’s biology; they thereby influence the child’s behavior, the quality of the child’s relationships, the child’s representations of self and others, and physical and psychological health over the lifespan (Nelson, 2013; Sroufe, 2013; Hanlon et al., 2020).

Building on these developments, recently developed explanatory models think about the phenomenon of gender dysphoria on multiple system levels—biological, relational, and cultural—and try to account for this complex interplay of factors. For example, in a hypothesis paper, Altinay and Anand propose that brain regions involved in self-body perception—what they call the *body image network*—show aberrant (lower) connectivity because of cumulative experiences that cause dissonance between subjective experience of gender and external feedback pertaining to “own-body and self” (gender) (Altinay and Anand, 2020). Whilst the authors focus exclusively on feedback pertaining to gender, negative external feedback about the self in the context of ACEs (including maltreatment) would also presumably modulate the body image network. In another hypothesis paper, Gliske (2019) proposes that changes in neural networks mediating distress, social behavior, and body ownership underpin the incongruence between the child’s subjective sense of gender and the sex assigned at birth. He further suggests that these changes in neural networks are, at least in part, the product of predisposing, precipitating, and perpetuating factors in the child’s relational, social, and cultural contexts (Gliske, 2019). Future research will need to test these hypothetical models and determine their utility and veracity.

In the gender dysphoria literature, research on the relational system level shows that supportive relationships contribute to better outcomes for children presenting with gender dysphoria. On coming out, a supportive response from family and peers promotes psychosocial well-being—including mental health—and reduces the degree of psychosocial impairment (Simons et al., 2013; Shiffman et al., 2016). Unfortunately, many children do not have a positive experience. Negative responses from family members, including outright rejection, are common, as is bullying by peers (Di Ceglie and Thummel, 2006; Specht et al., 2020; Strauss et al., 2020b).

More broadly, and separate from the responses to a child’s coming out, the available research—though sparse—suggests that some role is played by the quality of children’s relationships with attachment figures, the quality of the family environment, and the presence of adverse events that may have increased family stress or contributed to the bifurcations in the child’s developmental pathway. In a small sample of preschool boys with gender dysphoria (*n* = 22), and using the Strange Situation coded via the ABCD method (see Box 2), Birkenfeld-Adams (2000) reported a higher rate of insecure attachment patterns (16/22, or 73%)—dismissing, preoccupied, and disorganized/controlling—than in control boys from the normative population (11/20, or 55%) (Goldberg, 1997; Birkenfeld-Adams, 2000).^1^ Using a self-report methodology with preadolescent children, Cooper et al. (2013) looked at two dimensions of attachment insecurity (avoidant with mother, preoccupied with mother) and three dimensions of gender identity (gender typicality, gender contentedness, felt pressure for gender differentiation) (Cooper et al., 2013). Felt pressure for gender differentiation—a form of gender identity usually associated with problematic adjustment—was associated with both measures of attachment insecurity.

**Box 2. The ABCD and DMM classification systems: a brief overview.**

This text box provides a brief overview of the development of the ABCD and DMM models from the original work of Mary Ainsworth. For a visual representation of the development of the ABCD and DMM systems, see Figure 2.

**Mary Ainsworth and the ABC Model of Attachment**

In the 1960s, Mary Ainsworth identified three patterns of attachment in 11-month-old infants (Ainsworth and Wittig, 1969; Ainsworth et al., 1978): group A (*anxious-avoidant*), group B (*secure*), and group C (*anxious-ambivalent/resistant*). Infants classified into the B group (*secure*) used the attachment figure as a safe base from which to explore, protested on separation, and sought comfort from the attachment figure on reunion before returning to play. Infants classified into the A group (*anxious-avoidant*) did not exhibit distress on separation and ignored the attachment figure on reunion. Infants classified into the C group (*anxious-ambivalent/resistant*) showed distress on separation and were clingy and difficult to comfort on reunion. Type B attachment was termed secure attachment and type As and C as insecure attachment. The assessment of attachment in infants was based on Ainsworth’s Strange Situation Procedure, which was taped and analyzed using a behavioral/relational analysis.

**Mary Main and Judith Solomon: The ABCD Model of Attachment**

Mary Main and Judith Solomon expanded Ainsworth’s model by adding the D (*disorganized*) classification for children with behaviors that represented disruption to the Ainsworth patterns (Main and Solomon, 1986, 1990; Duschinsky and Solomon, 2017). The D category is also referred to as disorganized/disorientated and disorganized/controlling (Main and Solomon, 1986; Duschinsky and Solomon, 2017). The attachment system using these four groups is sometimes referred to as the ABCD system (Crittenden et al., 2010). Children classified into the D group were those that appeared to be so conflicted about approaching the attachment figure that they could not produce a coherent strategy when reunited (Landa and Duschinsky, 2013). Instead, they showed a broad range of odd behaviors that were termed *disorganized*. In the ABCD model, Type B attachment was termed secure attachment and Types A, C, and D as insecure attachment. In infancy and the preschool years, assessment of attachment using the ABCD model also involved the use of Ainsworth’s Strange Situation Procedure, which was taped and analyzed using a behavioral/relational analysis.

In school-age children, adolescents, and adults, attachment models derived from the ABCD model (Ammaniti et al., 2000; Main et al., 2003; Target et al., 2003; Giovanardi et al., 2018) continued to use the same four categories that were used in infants and preschoolers, but with a different terminology. Group A is called *dismissing* (given the shorthand of *Ds*; see Figure 2); Group B is called *secure* (given the shorthand of *F*; see Figure 2); group C is called *preoccupied* (given the shorthand of *E*; see Figure 2), and group D is called a *disorganized/unresolved state of mind or cannot classify* (given the shorthand of *U/CC*). Individuals classified as *secure* maintain a coherent narrative of their attachment history. Individuals classified as *dismissing* minimize the importance and impact of attachment relationships in their own lives. Individuals classified as preoccupied show an excessive preoccupation with attachment relationships. And individuals classified as disorganized/unresolved state of mind are coded as having unresolved loss or trauma (or have narratives that cannot be coded as falling into the three primary groups).

**Patricia Crittenden and the DMM Model**

Also building on Ainsworth’s original model, Patricia Crittenden developed an alternate system, the Dynamic-Maturational Model of Attachment and Adaptation (DMM). The DMM was developed with an emphasis on at-risk children and the self-protective (attachment) strategies that they used to promote maximal safety and comfort with attachment figures who are not sensitive or who are, in and of themselves, a source of danger (Crittenden, 1999). In this context the “disorganized” group within the ABCD system was reconceptualized as being organized in specific ways. The following high-risk patterns were identified: A3-4 and A5-6 (also known together as A+); C3-4 and C5-6 (also known together as C+) (Crittenden, 1999; Crittenden et al., 2010); and mixed A/C (see Figure 2).

In the DMM, risk is depicted along the arc of the model. Type B and low-subscript Type A and Type C strategies, found at the top of the arc, reflect normative, low-risk attachment strategies typical of non-clinical populations. High-subscript Type A and Type C strategies, and A/C, reflect high-risk attachment strategies typical of clinical or other high-risk populations.

In addition, the DMM models pays significant attention to the Type A and Type C developmental pathways, and sees them along a continuum of risk.

Children who develop along the Type A developmental pathway have attachment figures who are insensitive and predictably unresponsive to the child’s distress. In infancy, the child inhibits displays of negative affect and, instead, looks away from the attachment figure when distressed (anxious-avoidant attachment). In the preschool years, the child continues to inhibit displays of negative affect when uncomfortable or distressed but learns to please the attachment figure by engaging in preferred activities and by displaying positive affect when engaged in such activities (Type A1-2 strategies). In relational contexts that are not safe, the child may go on to develop a range of at-risk Type A attachment strategies. The at-risk Type A strategies involve inhibition of displays of negative affect coupled with displays of false-positive affect, idealization and exoneration of the parent, and blaming of the self, combined with caregiving to the attachment figure (A3 strategy, *compulsively caregiving*); compliance to the expectations and demands attachment figure (A4 strategy, *compulsively compliant*); seeking closeness to strangers and not attachment figures (A5 strategy, *compulsively promiscuous*); and seeking to protect themselves by relying on no one other than themselves (A6 strategy, *compulsively self-reliant*).

Children who develop along the Type C developmental pathway have attachment figures who respond inconsistently and unpredictably to the child’s signals of distress. In the preschool years, the capacity for disarming (coy) behaviors—which emerge from about 18 months of age—enables the child to use alternating displays of anger, fear, or disarming behaviors to help increase parental predictability and responsiveness (the Type C1-2 strategies). If the C1-2 strategies fail to elicit comfort and protection, the preschool/school-age child may increase the intensity of the affective displays. In Type C narratives, attachment figures are blamed, and the self is exonerated from any blame. Angry behavior escalates to aggression, and disarming behavior into feigned helplessness, both of which function to coerce attachment figures into responding (Type C3-4 strategies, *aggressive/feigned helpless*). At-risk older children may come to use the C5-6 strategies, where negative affect—fear, anger, or desire for comfort—continues to drive the strategy but is no longer signaled in an open and direct way (Type C5-6 strategies, *punitive/seductive*).

**Comparing the ABCD and DMM**

Despite the differences between the two systems, attachment patterns in both systems ABCD and DMM can be clustered by risk. Secure/B1-5 patterns are associated with well-being, with no risk. Dismissing/A1-2 and preoccupied/C1-2 are less comfortable but found in normative populations and are associated with low risk. Disorganized state of mind/A+ and C+ and A/C are high-risk categories associated with increased risk of distress, maladaptation, and psychopathology.

In adults with gender dysphoria, and using the Adult Attachment Interview (AAI) coded using the ABCD method in a larger sample (*n* = 95), Giovanardi et al. (2018) found that 27% adults with gender dysphoria (vs. 61% controls) were classified as secure. In those with insecure classifications 14% (vs. 19% controls) were dismissing, 13% were preoccupied (versus 7% controls) and 46% (vs. 13% controls) were classified as reflecting a disorganized/unresolved state of mind (Giovanardi et al., 2018). Key themes emerging from the attachment narratives were “a lack of autobiographical coherence and integration, revealing difficulties in connecting the past and present elements of one’s personal history, depicting balanced portraits of caregiving figures and giving importance to attachment experiences” (p. 8). In addition, it was noted that the gender dysphoria “group showed higher Anger toward fathers and, to a lesser extent (with smaller effect sizes), Derogation toward fathers and Idealization toward mothers, relative to controls” (p. 8).

Giovanardi et al. (2018) used both the AAI and the Complex Trauma Questionnaire to look at the comparative rates of ACEs in their adult subjects versus controls. Those with gender dysphoria reported significantly more ACEs, including mother-child relationships characterized by neglect, rejection, psychological abuse, and physical abuse, as well as domestic violence.

In order to examine rates of organized (secure, dismissing, and preoccupied) and disorganized (unresolved/disorganized) attachment, Giovanardi et al. (2018) also compared the gender dysphoria cohort to other normative and clinical samples from international and national meta-analyses. They found that, whilst adults with gender dysphoria differed from normative samples, they did not differ from other clinical samples.

The findings from the above-described studies cohere with the broader literature that suggests strong associations between quality of attachment and ACEs (Riggs, 2010), quality of attachment and psychiatric disorders (Carlson, 1998; Sroufe, 2005; Crittenden et al., 2010; Spruit et al., 2020), and ACEs and the presence of physical health and mental health issues across the life span (Felitti et al., 1998; Felitti, 2009; Flaherty et al., 2009; Shonkoff, 2016). Taken together, these studies suggest that gender dysphoria—like other physical and mental health problems—needs to be conceptualized in the context of the child’s lived experience, the lived experience of previous generations, and the many different ways in which lived experience is biologically embedded to shape the developing brain and to steer each child along their developmental pathway.

The goal of the current study is to examine the quality of parent-child attachment—also known as *patterns of attachment* (Ainsworth et al., 1978), *self-protective strategies* (Crittenden, 1999), or, simply, *attachment strategies*—in children presenting with dysphoria about their birth-assigned sex to a newly established, dedicated gender service at a tertiary care pediatric hospital. Based on previous studies with children, we hypothesized that school-age children and adolescents presenting for assessment and treatment of gender dysphoria would show an increase in at-risk patterns of attachment and higher rates of unresolved loss and trauma. We also hypothesized that on family assessment—which included the acquisition of a developmental history—children and families would report significant levels of family stress and ACEs that had adversely affected the health and well-being of the presenting child and the family.

## Materials and Methods

### Participant Recruitment

**FIGURE 1:**
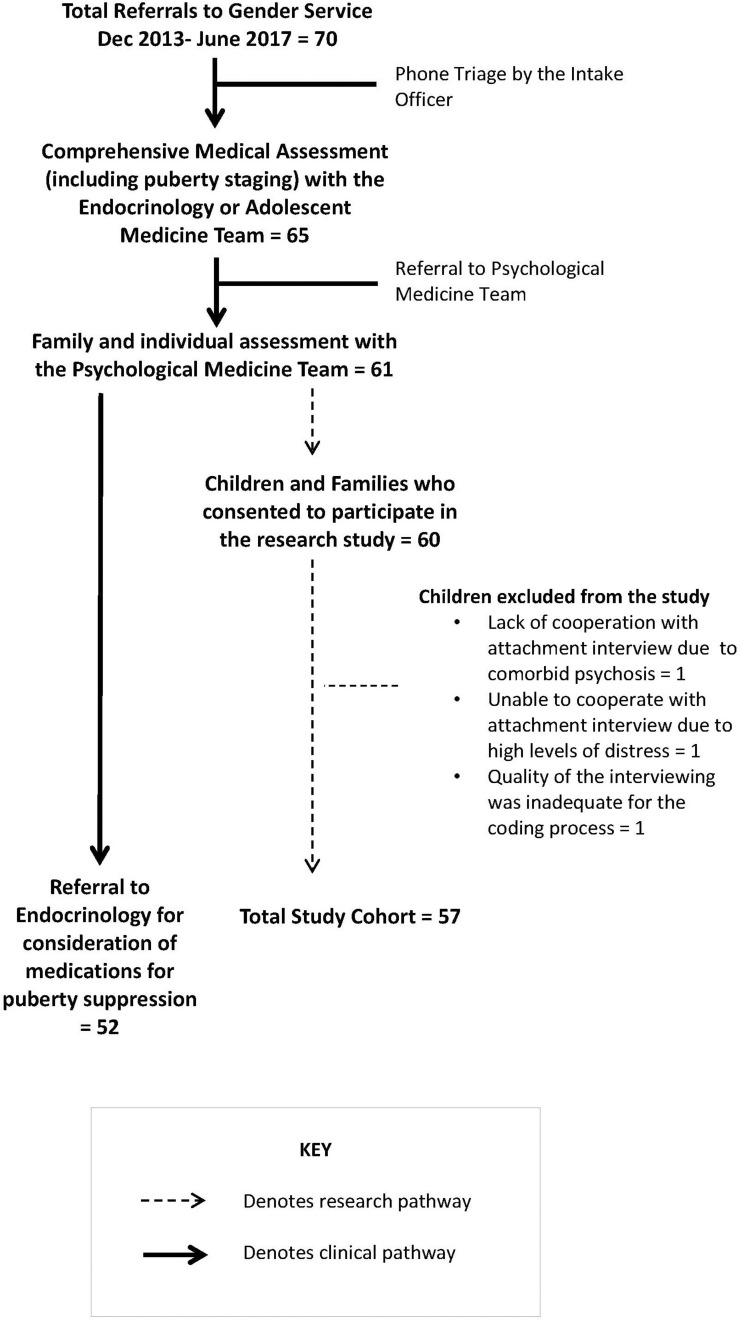
The assessment pathway within the multidisciplinary gender service.

During the period December 2013 to June 2017, 70 children experiencing dysphoria in relation to their birth-assigned sex were referred by their family doctors to a newly established—and not yet funded—Gender Service. Figure 1 summarizes the assessment pathway within the Gender Service.

### Procedure

**FIGURE 2:**
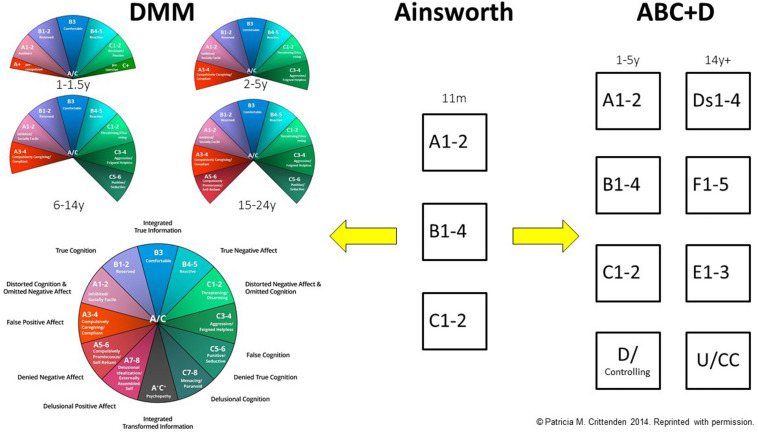
The ABCD and DMM classification systems. This figure is a visual representation of the development of the ABCD and DMM classification systems from Mary Ainsworth’s original work with 11-month-old infants, where she identified three patterns of attachment: A, B, and C (see **middle section**). Main and Solomon (1986, 1990) subsequently extended Ainsworth’s work by developing the ABCD model (see **right-hand side**). Patricia Crittenden (Crittenden, 1999) subsequently extended Mary Ainsworth’s work by developing the DMM model (see **left-hand side**). For more information see Box 2. In infancy and the preschool years: A1-2 = Group A, anxious-avoidant attachment; B1-4 = Group B, secure attachment; C1-2 = Group C, anxious-ambivalent/resistant attachment; D/controlling = disorganized/controlling (also known as disorganized/disoriented or just as disorganized). In late adolescence, and adulthood: Ds1-4 = Dismissing attachment; F1-5 = Secure; E1-3 = Preoccupied; U/CC = Unresolved state of mind/cannot classify (also known as disorganized/unresolved state of mind or cannot classify). © Patricia M. Crittenden 2014. Reproduced with permission.

The assessment process for gender dysphoria included participation in a comprehensive clinical assessment—including a family assessment and an individual assessment with the child—in the Department of Psychological Medicine (see Figure 1). The family assessment was a structured interview of 90–120 min duration that generated a three-generation genogram and a detailed developmental history of the child’s development in the context of the family and events that had affected the well-being of the presenting child and the family. The individual assessment provided the child with a therapeutic space in which they were able to share any additional information that had been difficult to share in the family session. For children (and families) who chose to participate in the research project, the assessments of attachment—developed as part of the Dynamic-Maturational Model of Attachment and Adaptation (DMM) (Crittenden, 2006)—were incorporated into the child’s individual assessment (see Figure 1). Children in primary school completed the School-Age Assessment of Attachment (SAA), and children in high school the Transition to Adult Attachment Interview (TAAI) (Farnfield et al., 2010). The SAA consists of cards whose themes address threats that school-age children frequently face or imagine facing. The interview protocol asks the child to make up a story about the child depicted on each card and then, if relevant, to recount a similar episode in the child’s own life. Each child was asked to choose whether the interviewer was to use the cards depicting a boy figure or a girl figure. The TAAI is a modified AAI. A narrative from the child is elicited through the early childhood memories of the parent-child relationship and episodes of loss or trauma. Modifications in the TAAI (relative to the AAI) include probes for affect-based memories and a wider range of potentially threatening circumstances, such as questions about peer relationships. The interviews were taped, deidentified, and transcribed, and were coded by blinded coders using the DMM coding system (see Box 2 and Figure 2).

### Other Measures

Alongside assessments of attachment, and based on the information provided by the child and family during the initial assessment, the Psychological Medicine team used various measurement tools and scales to document key aspects of the child’s relational context.

Socio-economic status was documented on the basis of the highest employment type—professional, white collar, blue collar, unemployed—of the parent(s) in the household that was the child’s primary residence.

The child’s global level of function was documented using Global Assessment of Functioning (GAF), a 100-point scale on which the 91–100 bracket denotes the highest level of functioning and the 1–10 bracket the lowest.

The child’s distress pertaining to gender was documented (by clinician consensus) on a four-point scale immediately following the clinical assessment process by the two clinicians (a child and adolescent psychiatrist and a clinical psychologist) who had delivered the assessment protocol. The four-point scale for distress pertaining to gender included: no distress (e.g., child feels well supported around the felt sense of gender issues/the developing body, and did not report any distress pertaining to their desire to be of the opposite gender), some distress (some distress around the felt sense of gender/the developing body), very distressed (substantial distress about puberty and body changes (e.g., not liking to touch the genitals when washing), and extreme distress (e.g., wanting to cut off body parts, hating the body, starving the body to remain prepubertal, self-harming because of hate for the body).

Each child’s distress pertaining to their life in general—which captured the child’s general sense of well-being in the family, with peers, and in the school contexts—was documented (also by clinician consensus) on a four-point distress scale.

Children who fell into the upper category on either scale required the immediate implementation of a safety plan and referral to local mental health services (if not already engaged). For description of the Traffic Light Safety Plan used with the children, see Online Supplement 16.2 in Kozlowska et al., 2020).

The family’s level of functioning was documented (also by clinician consensus) using four categories: harmonious, some conflict, high conflict, and harmonious but experiencing stress in the context of a current major life stress.

Reported ACEs, DSM-5 diagnoses, and the child’s situation with regard to close relationships with peers (their social connectedness with peers) were also documented.

### Comparison Groups

#### Non-clinical Comparison Group

Non-clinical children from the same catchment area were matched for age and biological sex and were recruited from wide-ranging SES. Non-clinical children were recruited from a variety of sources—children of hospital employees (cleaners, parking attendants, administration staff, security staff, and health care professions) and their friends from school, youth clubs, soccer teams, and so on. Country children were recruited from families of administration staff in holiday accommodation agencies (as well as their friends). Children were excluded from the non-clinical comparison group if they or a close family member (mother, father, sister, or brother) had a previous DSM diagnosis, if they or a close family member was attending a counseling or other psychological service (or if such a service was being sought), or if they did not speak English, had a family history of genetic disorders, or suffered from a chronic medical illness or brain injury. Non-clinical children in primary school completed the SAA, and children in high school the TAAI (Farnfield et al., 2010). After completing the attachment interview with the child, the interviewer (in a discussion with the child and parent) used a checklist to document ACEs. The checklist included the same items—adverse life experiences—that were screened for in the clinical assessment with children with gender dysphoria and their families: illness events (for the child), maternal mental illness, maternal physical illness, paternal mental illness, paternal physical illness, family conflict, loss events (death), loss events (separation), bullying, multiple moves of house, migration, financial stress, custody battle, physical abuse, sexual abuse, emotional abuse, neglect, domestic violence, and other.

#### Mixed Psychiatric Comparison Group

The second comparison group comprised 51 school-age children with mixed psychiatric diagnoses who had been referred to the same hospital department some years earlier (with results published in a previous study, Crittenden et al., 2010).

### Ethics Statement

The study was approved by the Hospital Ethics Committee. Participants and their legal guardians provided written informed consent in accordance with national health and medical research council guidelines.

### Data Analysis

Transcripts of the SAA and TAAI were coded by blinded coders using the DMM methodology. The DMM coding process yielded three pieces of information that were used to compare the gender dysphoria group against the non-clinical comparison group and mixed psychiatric comparison group.

– The pattern of attachment/self-protective strategy reflecting not only the child’s habitual way of obtaining comfort and protection from attachment figures but also the information processing underlying the pattern. The low-subscript strategies (A1-2, B1-5, and C1-2) are normative strategies associated with health and well-being and are seen commonly in the normative population. The high-subscript strategies—characterized by increased errors in information processing and by less coherence—are at-risk strategies associated with stress, distress, and psychopathology, and are seen commonly in clinical populations.
– Unresolved loss and trauma, which reflect particular dangers or losses that had generated extreme psychological efforts to protect the self, show themselves in the narrative by linguistic dysfluencies regarding particular life events.
– Modifiers of the self-protective strategy refer to linguistic markers that suggest that the self-protective strategy is not working for the child. The depression modifier—reported in this study—refers to the presence of continuous low arousal that makes strategic action seem futile. Modifiers “reflect both the child’s inability to discover an effective self-protective strategy and also [the child’s] continued attempt to use one; it is rather like continuing to hit a locked door with a hammer—long after one discovers that the hammer is useless” (p. 193) (Crittenden et al., 2010).

Chi square tests and *t*-tests were used to analyze categorical and continuous variables, respectively. For between-group chi square analyses of attachment pattern/self-protective strategies, the strategies were grouped by risk (Crittenden et al., 2010): low risk (B1, A1-2, C1-2); moderate risk (A3-4, C3-4); and high risk (A5-6, C5-6, A/C). For analysis of the depression modifier, children who met full criteria for the modifier and those who met partial criteria were subsumed into a single group.

In a whole-group analysis, a logistic regression was run to ascertain whether children with higher levels of loss or trauma or whose DMM coding, as described above, included the depression modifier were more likely to be classified into the at-risk attachment patterns.

Qualitative analyses included a visual representation of self-protective strategies to examine the pattern of results and to identify clusters (developmental pathways). Key themes for loss and trauma via the linguistic analysis used by the blinded coders were also identified.

Within the gender dysphoria group, *post hoc* chi square analyses examined whether there were any differences in patterns of attachment between school-age children and adolescents, between children with preschool vs. later onset, and between biological males and biological females (sex assigned at birth). Also within the gender dysphoria group, *post hoc* analyses—chi square analyses for categorical data and *t*-tests for continuous data—were run to examine the potential contribution of different risk factors to attachment status: age, biological sex, SES, parent marital status (biological parents vs. other family constellation), toddler vs. later onset, gender distress, general life distress, level of family function, quality of self-reported peer relationships, mental health score (sum total of diagnoses and other mental health variables), ACE score, and maltreatment score (sum score of ACEs that fall under the umbrella of maltreatment). Because numbers in the low-risk group were very small, for this exploratory analysis the children were collapsed into two groups: a low- and moderate-risk group [B1, A1-2, C1-2, A3-4, C3-4] (*n* = 20) and a high-risk group [A3-4(5-6), C3-4(5-6), A5-6, C5-6, A/C] (*n* = 37).

Using the above-described between-group and within-group analyses as a guide, a binary logistic regression was performed across the cohort as a whole to investigate the effects that age, sex, SES, family constellation (biological parents vs. other family constellation), and ACEs (including maltreatment) had on attachment risk: low-risk attachment group (B1, A1-2, C1-2) vs. moderate- and high-risk attachment group (all other attachment patterns). Neither unresolved loss and trauma nor the depression modifier was included in this analysis because these components of the attachment classification overlap with the information about adverse events (including maltreatment) represented in the total ACE score.

## Results

### Participant Characteristics

The final sample comprised 57 children/adolescents aged 8.42–15.92 years (mean = 12.96; *SD* = 1.91; median = 13.67) presenting with feelings of dysphoria about the sex that had been assigned to them at birth. On chromosomal testing, 24 (42.1%) were XY “biological males,” and 33 (57.9%) were XX “biological females” (all of which matched their sex assigned at birth). Thirteen (23.8%) were prepubertal (Tanner stage 1), 36 (63.2%) were pubertal (Tanner stages 2–4), and 5 were post-pubertal (Tanner stage 5).

Of the birth-assigned males, 21/24 children experienced their gender as female—being a girl or wanting to be a girl. One child wanted to be of a neutral gender; one wanted to be both genders at the same time; and one experienced intrusive thoughts about gender and wanted to be a girl during bouts of depression but not when euthymic. Of the birth-assigned females, 31/33 biological experienced their gender as male—being a boy or wanting to be a boy. One child liked to wear male clothes, wanted to be “[child’s name] in boys’ clothes,” but had never expressed the desire to be a boy in words. One child was distressed about gender but was unable to clearly express relevant feelings and thoughts.

At the time of assessment, the children’s levels of distress pertaining to gender and to their overall well-being and life situations were high (see Table 1). On DSM-5 criteria, 47 (82.5%) children met criteria for a formal gender dysphoria diagnosis (see Table 2). Comorbid psychiatric diagnoses (*n* = 50; 87.7%), as well as histories of self-harm, suicidal ideation, and symptoms of distress, were common (see Table 2).

**TABLE 1:** Self-reported distress and quality of the child’s relational context as reported by the child and family during the psychological medicine assessment.

Self-report dimension				
**Child’s level of distress pertaining to gender assigned at birth**	**No distress** 0 (0.00%)	**Some distress** 14 (24.6%)	**Very distressed** 28 (49.1%)	**Extreme distress** 15 (26.3%)
**Child’s level of distress pertaining to general well-being in the family, peer, and school settings**	**No distress** 6 (10.5%)	**Some distress** 16 (28.1%)	**Very distressed** 20 (35.1%)	**Extreme distress** 15 (26.3%)
**Levels of function reported by the family**	**Harmonious** 14 (24.6%)	**Some conflict** 22 (38.6)	**High conflict** 16 (28.11%)	**Harmony disrupted by major life event** 5 (8.8%)
**Quality of self-reported peer relationships at assessment**	**More than one close friend** 39 (68.4%)	**One close friend** 4 (7%)	**No close friend currently or negative peer relationships** 6 (10.5%)	**No close friends ever** 8 (14%)

**TABLE 2:** Clinical information about participants with FND from clinical assessment.

Clinical information, diagnoses and comorbid symptoms	Number (total *n* = 57)	Percentage
**Psychiatric diagnoses (DSM-5)**		
Gender Dysphoria code 302.6 (F64.2) or 302.85 (F64.1)	47	82.5%
Other Specified Gender Dysphoria code 302.6 (F64.1)	3	5.3%
Unspecified Gender Dysphoria code 302.6 (F64.8)	4	7.0%
Does not meet criteria for the above	3	5.3%
Comorbid mental health diagnosis (DSM-5)	50	87.7%
Anxiety	38	66.7%
Depression	36	63.2%
Any behavioral disorder	21	36.8%
Autism	9	15.8%
Learning disorder	8	14%
Other symptoms relevant to mental health		
Self-harm occurring in the present	7	12.3%
Self-harm history	30	52.6%
Suicidal ideation	28	49.1%
Suicide attempt	6	10.5%
Child Protection Services involvement	12	21%
**Adverse childhood experiences (ACEs)**		
Family conflict	38	66.7%
Loss via separation from a loved one or a close friend	34	59.6%
Bullying	34	59.6%
Maternal mental illness (most commonly depression)	30	52.6%
Paternal mental illness	23	40.4%
Financial stress	21	36.8%
Moving house that had been stressful	16	28.1%
Domestic violence	14	24.6%
Maternal physical illness	12	21.1%
Physical abuse	11	19.3%
Sexual abuse	6	17.5%
Placement changes (foster care or between parents)	7	12.3%
Neglect	6	10.5%
Custody battle	6	10.5%
**Intelligence quota estimated from school testing and school reports**		
Superior range (120+)	11	19.3%
Average range (80–119)	40	70.2%
Borderline range (70–79)	6	10.5%

Difficulties in peer relationships were a common theme in the assessment interviews, and 34 (59.6%) children had experienced bullying (see Table 2). Nonetheless, three-quarters of participants reported that they had one or more close friends (see Table 1).

As described by children and their families, the ages at which children discovered or disclosed their gender dysphoria largely fell into four categories: (1) children who had expressed their gender preferences in words, behavior (e.g., refusal to wear dresses), and play from the preschool years onward (*n* = 32; 56.1%); (2) children who dated their feelings of gender dysphoria from the school-age years (*n* = 14; 24.6%); (3) children who dated these feelings from the prepubertal time period, when awareness that their own puberty was approaching had triggered their distress (*n* = 8; 14.0%); and (4) children who dated these feelings from the post-pubertal period (*n* = 3; 5.3%). Age of disclosure in the four subsets, respectively, was 2.5–14 years (mean = 9.12 years); 8–13.8 years (mean = 11.49), 12.5–14.5 years (mean = 13.34), and 12.0–14.8 years (mean = 13.61). Most of the children had confided feelings of gender dysphoria to attachment figures: mother (*n* = 28, 49.1%); father (*n* = 3; 5.3%); and both parents/other family members (*n* = 7; 12.3%).

Children from the non-clinical comparison group—matched on age and biological sex—included 24 *cis* boys and 33 *cis* girls (9–16.08 years; mean = 12.83; *SD* = 2.01; median = 13.00). The mixed psychiatric comparison group comprised 51 school-age children with mixed psychiatric diagnoses, 29 *cis* boys and 22 *cis* girls (5–12 years; mean = 9.63; *SD* = 1.82; median = 10.00) who had been referred to the same hospital department some years earlier (with results published in a previous study, Crittenden et al., 2010).

### Family Characteristics of Children With Gender Dysphoria

Families spanned all socioeconomic classes: professional (*n* = 7; 29.8%), white collar (*n* = 19; 33.3%), blue collar (*n* = 16; 21.8%), and unemployed (*n* = 5; 8.8%). Children lived in a variety of family settings: biological parents (*n* = 20; 35.1%); a biological mother who had re-partnered (*n* = 9; 15.8%); a biological father who had re-partnered (*n* = 3; 5.3%); a biological parent, usually the mother (*n* = 22; 38.6%); and foster care (*n* = 3, 5.3%). Forty-seven (82.5%) had one of more siblings as part of their family units. Ten children (17.5%) reported rejection—related to their gender preference—via a family member: father (*n* = 4), mother, (*n* = 3), or sibling (*n* = 5). Levels of family stress were high (see Table 1).

### Between-Group Comparisons on Demographics and ACEs

Comparisons between the gender dysphoria and non-clinical comparison group confirmed that the children were matched on biological sex (χ^2^ = 0; *p* = 1) and age [*t*(112) = 0.334; *p* = 0.739]. The groups were also comparable on family socioeconomic status (χ^2^ = 0.47; *p* = 0.93).

As noted above, only 20, or 35%, of the children with gender dysphoria lived in a traditional family unit made up of a biological mother and father, whereas 75% of the children in the non-clinical group lived with their biological mothers and fathers (χ^2^ = 18.76; *p* < 0.001). Children with gender dysphoria—and their families—reported more ACEs than children in the non-clinical group [mean = 5.5 vs. 1.7; *t*(76.37) = 7.81; *p* < 0.001] (see Table 2).

### Levels of Functional Impairment on the GAF

On the GAF, children with gender dysphoria scored into functional categories that reflected a loss of health and well-being (range 25 − 95/100; mean = 54.88; *SD* = 15.40; median = 55.00) (see Table 3).

**TABLE 3:** Global assessment of function scores in the 57 children with gender dysphoria.

Category	Descriptions	Score	Number	Percentage
**Superior in all areas**	No symptoms; physically able; excellent relationships with family and friends; wide range of extracurricular activities; doing well at school/preschool; developing normally; everyday problems never get out of hand.	91–100	1	1.8%
**Good in all areas**	Virtually no symptoms; usually copes well; physically able; good relationships; normal play and leisure activities; school/preschool OK; may have problems when stressed but these are short-lived and only occasionally get out of hand.	81–90	3	5.3%
**No more than slight problems**	Some significant symptoms, only briefly get out of hand; sometimes child gets distressed; short-term or little interference with mobility or relationships or play and leisure activities; school/preschool may be slightly affected or affected for a short time.	71–80	4	7.0%
**Some difficulty in a single area but generally doing pretty well**	Mild symptoms that recover quickly with treatment; any distress or disability does not stop child doing most things done at that age; some anxiety or irritability or brief mood changes; minor effect on mobility or school/preschool or relationships or play and leisure activities; problems may persist but may only be recognized by those who know the child.	61–70	12	21.1%
**Variable problems in some but not all areas**	Moderate symptoms have significant disabling effect on child; minor to moderate effect on mobility; school/preschool may be affected; relationships or play and leisure activities may be affected; may need special education; in some situations may seem OK; mainly managed in outpatient clinic or by family doctor.	51–60	13	22.8%
**Severe problems in one area OR moderate problems in most areas**	Severe symptoms having a major effect on child’s life; restricted mobility; relationships or play and leisure activities are affected; child is distressed or has difficult behavior; some relationships are maintained; learning difficulties or problems with or missing school; likely to have been seen by specialist.	41–50	14	24.6%
**Major problems in several areas AND unable to function in one of these areas**	Severe, almost constant symptoms; child is distressed, withdrawn, or has strange or aggressive behavior; significant limitations on mobility or school/preschool or relationships or play and leisure activities; specialist management needed.	31–40	9	15.8%
**Unable to function in almost all areas**	Very severe symptoms; child is very distressed; likely to be confined to bed; unable to go to school/preschool; may be in hospital but child is not entirely dependent on others.	21–30	1	1.8%
**Needs nursing supervision**	Confined to bed; in hospital; very severe symptoms but stable; needs help with self-care, which a child the same age can do without help.	11–20	0	0%
**Needs constant supervision**	High (24-h) medical dependency (e.g., in intensive care unit); life-threatening symptoms, including suicidal/homicidal risk.	1–10	0	0%

### Intercoder Reliability on Patterns of Attachment/Self-Protective Strategies

All deidentified SAAs and TAAIs were coded by blinded coders trained in DMM discourse analysis. Intercoder agreement between coder 1 (coded both SAAs and TAAIs) and coder 2 (coded TAAIs only) was 85% (κ = 0.821; *p* = 0.001) with regard to self-protective strategies (Types A5-6, A3-4, A1-2, B1-5, C1-2, C3-4, C5-6, and A+/C+) and 100% when the patterns were collapsed into the four relevant clusters (Types A+, C+, and A+/C+, plus normative/balanced). Intercoder agreement between coder 1 and coder 3 (coded SAAs only) was 70% (κ = 0.565, *p* = 0.001) with regard to self-protective strategies (Types A5-6, A3-4, A1-2, B1-5, C1-2, C3-4, C5-6, and A+/C+) and 100% when these patterns were collapsed into the four relevant clusters (Types A+, C+, and A+/C+, plus normative/balanced).

### Comparisons on Patterns of Attachment/Self-Protective Strategies

In contrast to children in the non-clinical group, who were mostly classified into the normative attachment strategies (A1-2, B1-5, and C1-2), children with gender dysphoria were mostly classified into the at-risk attachment strategies (A3-4, A5-6, C3-4, and C5-6) (χ^2^ = 52.66; *p* < 0.001) (see Table 4).

**TABLE 4:** Comparison of attachment sub-patterns by risk.

Attachment sub-patterns by risk	Gender dysphoria group	Non-clinical group	Mixed psychiatric disorder group (Crittenden et al., 2010)
No risk (B, A1-2, C1-2)	8 (14.0%)	47 (82.5%)	3 (6%)
Moderate risk (A3-4, C3-4)	30 (52.6%)	7 (12.3%)	27 (53%)
High risk (A5-6, C5-6, A/C)	18 (33.3%)	3 (5.3%)	21 (41%)
Total	57	57	51

**FIGURE 3:**
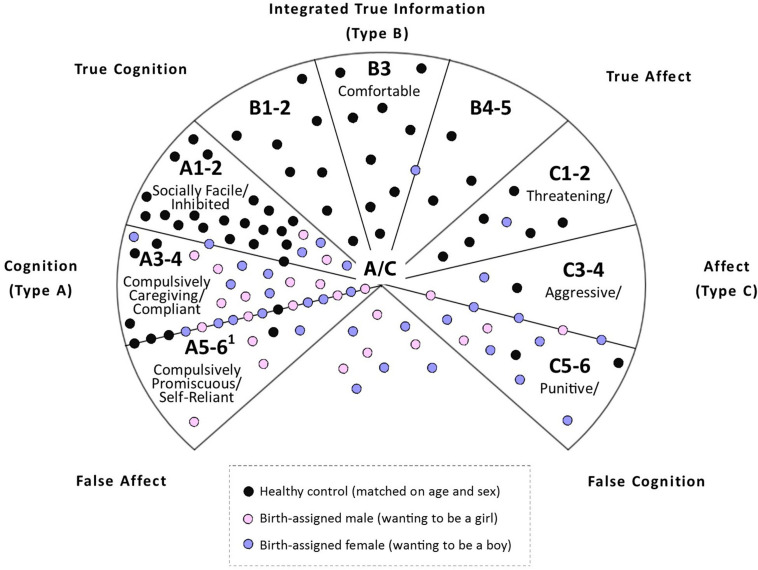
A dynamic-maturational model of patterns of attachment in school age and adolescence. The model depicts patterns of attachment in School Age and Adolescence. Low subscript patterns depicted in the top part of the circle (A1-2; B1-5; and C1-2) reflect normative patterns of attachment. High subscript patterns depicted in the bottom part of the circle (A3-4; A5-6; C3-4; C5-6, and A/C) reflect at-risk patterns of attachment that are more commonly found in clinical populations. © Patricia M. Crittenden 2001. Reproduced with permission.

A visual representation of the results—depicted in Figure 3—shows that children with gender dysphoria fell into four clusters or developmental pathways. The first cluster of children (*n* = 8; 14%) used normative attachment strategies, which enable children to seek comfort and protection from their attachment figures when faced with significant distress or in situations of danger. The second cluster of children (*n* = 31; 54.3%) used Type A+ strategies (Types A3-6), in which children inhibit expression of negative affect and comply with parental expectations to maximize the approval, closeness, and protection received from attachment figures (see also section “Discussion”). An unexpected finding within this group was the high proportion of children—17/31 in this cluster—whom the blinded coders classified as Type A6 (self-reliant) or documented elements of the Type A6 strategy in brackets, alongside the lower-subscript Type A3-4 strategy [therefore generating the classification A3-4(6)]. The third cluster of children (*n* = 10; 17.5%) used the C5 strategy, in which children control the expression of anger and use deception in an effort to protect the self and put blame on others. A subgroup of these children (5/10) were coded as *triangulated C5* because the narratives included denigration of one parent and idealization of the other—termed *triangulation* in the terminology of family therapy (see also section “Discussion”). The fourth cluster of children (*n* = 8; 14%) used Type A/C strategies, in which children used Type A+ strategies with one attachment figure or in a certain context and Types C+ strategies with another attachment figure or in a different context. Of these children, 6/8 used the C5 strategy—*triangulated C5* in all 6 cases—as part of the A/C strategy. When these children are taken into account, the overall number of children using the C5 strategy increased to 16 (28% of all participants) and the *triangulated C5* subgroup to 11 (19% of all participants).

### Comparisons Between Groups on Unresolved Loss and Trauma

On linguistic analysis, children with gender dysphoria had a high rate of unresolved loss and trauma compared to children in the non-clinical group (χ^2^ = 18.64; *p* < 0.001) (see Table 5). The most common themes noted by the blinded coders were bullying or peer issues related to gender identity (*n* = 8), family conflict (*n* = 6), lack of protection, or unstable behavior, by the attachment figure (*n* = 5), loss events (by death or separation) (*n* = 5), maltreatment (*n* = 4), domestic violence (*n* = 3), rejection by father (*n* = 3), abandonment by attachment figure (*n* = 1), and mother’s suicide attempt (*n* = 1).

**TABLE 5:** Unresolved loss or trauma.

Unresolved loss of trauma	Gender dysphornia group	Non-clinical group	Mixed psychiatric disorder (Crittenden et al., 2010)
Not coded	26 (45.6%)	48 (84.2%)	18 (35.3%)
Coded	31 (54.4%)	9 (15.8%)	30 (64.7%)

### Comparisons Between Groups on the Depression Modifier

On linguistic analysis, children with gender dysphoria had a high rate of the depression modifier compared to children in the non-clinical group (χ^2^ = 22.60; *p* < 0.001) (see Table 6).

**TABLE 6:** Rates of the depression modifier.

Depression modifier	Gender dysphoria group	Non-clinical group	Mixed psychiatric disorder group (Crittenden et al., 2010)
Not coded	34 (59.6%)	55 (96.5%)	22 (43.1%)
Coded	23 (40.4%)	2 (3.5%)	29 (56.9%)

### Contribution of Unresolved Loss and Trauma and the Depression Modifier to Attachment Risk

In an effort to determine whether unresolved loss and trauma and the depression modifier—classified by blinded coders via linguistic analysis—increased attachment risk (low-risk attachment vs. moderate- and high-risk attachment) across the cohort as a whole, we conducted an analysis using a binary logistical regression model; the analysis yielded significant results [χ^2^(2) = 41.96; *p* ≤ 0.001]. It explained 41% (Nagelkerke *R*^2^) of the variation in attachment risk, with 77.2% of cases correctly classified. Children were 4 times more likely to be classified into the moderate- and high-risk attachment group for every identified event of unresolved loss and trauma. Likewise, children who were given a depression modifier were 22 times more likely to also be classified into a moderate-to-high risk attachment pattern. The statistics for the depression modifier should be interpreted with caution, however, because of the small sample of children with the depression specifier (*n* = 22) and also the variability indicated by the confidence intervals (see Table 7).

**TABLE 7:** Logistic regression looking at the contribution of unresolved loss and trauma and the depression modifier to attachment risk (classification into the moderate- and high-attachment group vs. the low-risk attachment group).

	Variable	Chi-square	df	*P*-value	Nag. R	−2LL	β	SE	Wald	df	*P*-value	Exp(B)	LCI	UCI
Step 0	Constant						0.070	0.187	0.140	1	0.708	1.073		
Step 1		41.96	2	*p* < 0.001	0.41	115.94								
Homer and Lemeshow Test		2.458	3	0.483										
	Unresolved loss or trauma						1.371	0.441	9.692	1	0.002	3.941	1.662	9.344
	No depression modifier applied (ref.)													
	Depression modifier applied						3.108	1.065	8.518	1	0.004	22.381	2.776	180.458

*df, degree of freedom; Nag. R, Nagelkerke R square; -2LL, -2 log likelihood; β, beta coefficient; SE, standardized error; Wald, Wald chi-square; Exp(B), odds ratio; LCI, lower confidence interval; UCI, upper confidence interval.*

### Comparisons Between the Gender Dysphoria Group and the Mixed Psychiatric Group

Whilst attachment strategies used by children with gender dysphoria differed significantly from those of children in the non-clinical group (see above), they did not differ from the cohort of school-age children with mixed psychiatric disorders (the mixed psychiatric group) (χ^2^ = 2.43; *p* = 0.30) (see Table 4). Likewise, there were no differences between these two groups in the rate of unresolved loss and trauma (χ^2^ = 0.70; *p* = 0.40) or in the rate of the depression modifier (χ^2^ = 2.94; *p* = 0.09) (see Table 6).

### *Post hoc* Analyses Within the Gender Dysphoria Group

Within the gender dysphoria group, there were no differences in patterns of attachment between school-age children and adolescents (χ^2^ = 5.11; *p* = 0.78), between children with preschool vs. later onset (χ^2^ = 1.85; *p* = 0.40), or between biological males and biological females (sex assigned at birth) (χ^2^ = 1.19; *p* = 0.55).

Within the gender dysphoria group, exploratory analyses looking at potential risk factors—low- and moderate-risk attachment group vs. moderate- and high-risk attachment group—showed that children in the higher-risk group were more likely to come from a lower SES (unemployed or blue collar) (χ^2^ = 9.99; *p* = 0.02) and to have experienced maltreatment (physical abuse, sexual abuse, emotional abuse, neglect, and exposure to domestic violence) [*t*(54.06) = −2.28; *p* = 0.027]. All other comparisons were non-significant.

### Binary Logistic Regression Looking at Psychosocial Factors Contributing to Attachment Risk

We also used the binary logistical regression model to investigate the effects that age, sex, SES, family constellation, and ACEs had on attachment risk (low-risk attachment vs. moderate- and high-risk attachment) across the cohort as a whole (children with gender dysphoria and children in the non-clinical group). The analysis yielded significant results [χ^2^(7) = 47.08; *p* < 0.001], explaining 45% (Nagelkerke *R*^2^) of the variation in attachment risk, with 79.8% of cases correctly classified. Based on these results, a significant positive association was identified between family constellation and attachment risk, with children living in a non-traditional family constellation being three times more likely to be classified into the moderate- and high-risk attachment group. Likewise, a significant positive association between ACEs and attachment risk was identified, indicating that for every additional ACE, the probability of the child being classified into the moderate-to-high risk attachment group increased 1.6 times. A significant negative association was also identified between age and attachment risk, indicating that for every year that age decreased, the probability that the child would fall in the moderate- and high-risk attachment group increased by 0.8 times. Because the gender clinic was set up for young children—prepubertal children and children in early adolescents—this association is likely to reflect referral bias. All other included variables were non-significant (see Table 8).

**TABLE 8:** Logistic Regression looking at the contribution of psychosocial factors to attachment risk (classification into the moderate-to-high attachment group vs. low risk attachment group).

	Variable	Chi-square	df	*P*-value	Nag. R	−2LL	β	*SE*	Wald	df	*P*-value	Exp(B)	LCI	UCI
Step 0	Constant						0.070	0.187	0.140	1	0.708	1.073		
Step 1		47.08	7	*p* < 0.001	0.45	110.82								
Homer and Lemeshow Test		12.463	8	0.132										
	Age						–0.267	0.134	3.967	1	0.046	0.766	0.589	0.996
	**Sex**													
	Male (ref.)													
	Female						0.124	0.535	0.053	1	0.817	1.132	0.396	3.231
	SES													
	Unemployed (ref)								4.406	3	0.221			
	Blue						1.633	0.951	2.952	1	0.086	5.121	0.795	33.006
	White						0.411	0.845	0.236	1	0.627	1.508	0.288	7.908
	Professional						1.202	0.940	1.634	1	0.201	3.325	0.527	20.908
	Family constellation													
	Biological (ref.)													
	Other						1.105	0.548	4.065	1	0.044	3.019	1.031	8.838
	ACE total						0.498	0.548	15.353	1	0.000	1.646	1.283	2.112

*df, degree of freedom; Nag. R, Nagelkerke R square; -2LL, -2 log likelihood; β, beta coefficient; SE, standardized error; Wald, Wald chi-square; Exp(B), odds ratio; LCI, lower confidence interval; UCI, upper confidence interval.*

## Discussion

In this study, we used DMM linguistic analysis of attachment interviews to examine patterns of attachment/self-protective strategies in children presenting with gender dysphoria and non-clinical children matched on age- and biological sex, and drawn from a broad range of SES. Whilst children from the non-clinical group were mostly classified into the normative attachment strategies, children with gender dysphoria were mostly classified into the at-risk attachment strategies. Children with gender dysphoria were also found to have high rates of unresolved loss/trauma and high rates of the depression modifier. The themes pertaining to unresolved loss/trauma identified by blinded coders were consistent with the developmental histories—and high rates of ACEs (including maltreatment)—reported by the children and their families during the family assessment process. Many families had experienced instability, conflict, parental psychiatric disorder, financial stress, maltreatment events, and relational ruptures that, in a small number, included rejection or abandonment of the presenting child by a father, sibling, or mother. For the young person presenting with gender dysphoria, these ACEs were compounded by experiences of bullying—which were common—and by the difficulties that the children described in their narratives of finding and maintaining close friendships. Within the gender dysphoria group, children coming from socioeconomically disadvantaged families and children who had experienced maltreatment (physical abuse, sexual abuse, emotional abuse, neglect, and exposure to domestic violence) were more likely to be classified into at-risk attachment patterns. In line with previous reports, the study also showed that comorbid psychiatric diagnoses were also common, as were histories of self-harm, suicidal ideation, and symptoms of distress.

The data from this study suggest that the developmental pathways of children with gender dysphoria—reflected in patterns of attachment—are shaped, at least in part, by ACEs (including maltreatment), loss of family stability and cohesion, and socioeconomic disadvantage. These finding cohere with the broader literature that shows associations between ACEs and health and well-being (inverse), ACEs and SES (inverse), and SES and health and well-being (positive) (Barnett et al., 2012; Allen et al., 2014; Kerker et al., 2015; Donkin et al., 2018; Hanlon et al., 2020). Whilst these associations are well established in the published literature, the causal pathways are complex and non-linear, and the processes and mechanisms by which adversity affects brain and body development, as well as the well-being of children and adolescents, are still in the process of being elucidated (Dudley et al., 2011; Barnett et al., 2012; Babenko et al., 2015; Shonkoff, 2016; Dagan et al., 2018; Agorastos et al., 2019; Rasmussen et al., 2019; Hanlon et al., 2020).

**FIGURE 4:**
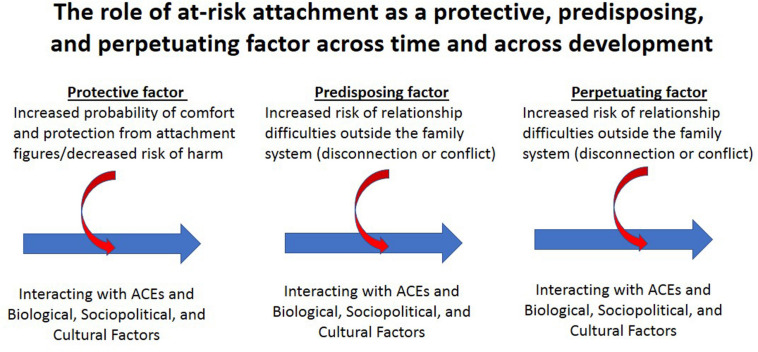
A visual representation depicting the role of at-risk attachment across time and development, along with the biological embedding of experience. Adapted from a figure by David L. Perez. © David L. Perez 2020. Reproduced with permission.

Nonetheless, from the perspective of attachment theory and systems thinking, the problems of troubled children, including those with gender dysphoria, emerge from a complex and ongoing interplay between genetic factors, experience (including ACEs and the quality of the child’s attachment relationships), the biological embedding of experience via epigenetic and neuroplastic mechanisms in the body and brain, and sociopolitical and cultural factors (including socioeconomic disadvantage) (Bowlby, 1973a, 1988). This interplay occurs across time in an ongoing fashion beginning with the previous generation(s), across the child’s lifespan—from conception to death—and into subsequent generations (see Figure 4). In this context the child’s pattern of attachment functions in a number of ways across development. Early in development the pattern of attachment is adaptive. It reflects the child’s effort to organize self-protectively in response to the child’s specific relational context to increase the probability of obtaining comfort and protection from attachment figures and, for some children, to protect the child from attachment figures who are dangerous. Later in development, at-risk attachment strategies becomes a risk factor for subsequent distress, psychopathology, and maladaptive development. Risk is conferred because, in large part, what was adaptive earlier in development—in the specific relationship with the child’s own parents—may be less so in other relationships, both within and outside the family. In this way at-risk attachment is initially protective but with time becomes both a predisposing and a perpetuating factor for distress and compromised development (see section “Discussion” of the high-subscript attachment strategies below) (see Figure 4).

The similarities between the results of the current study and those by Giovanardi et al. (2018) in adults with gender dysphoria are striking. Despite the use of two different classification systems—the ABCD system in the adult study versus the DMM in this one (see Figure 2 and Box 2)—both studies found that only a small percentage of narratives were classified into the normative attachment patterns (dismissing, secure, and preoccupied in the ABCD system and A1-2, B1-5, and C1-2 in the DMM system). Instead, a large percentage of narratives were classified into high-risk attachment patterns (disorganized/unresolved state of mind in the ABCD system and A3-4/A5-6, C3-4/C5-6 and A/C in the DMM system). On linguistic analysis, the high-risk patterns are characterized by a lack of coherence: the speaker is unable to provide a coherent narrative of their attachment relationships (referred to as lack of coherence). In the DMM methodology, this lack of coherence includes the following: bias in information processing (Crittenden and Heller, 2017); inconsistencies between various memory systems; recurring linguistic dysfluencies, along with unregulated fragments of distressing thoughts, feelings, and memories, around specific loss or trauma events; and, in some cases, a pervasive sense of futility because the strategy was not working, with the consequence that the children were struggling to meet their basic needs for parental comfort and protection.

A particular strength of the DMM—and the information-processing methodology used to code transcripts—is that the category of “disorganized” in the ABCD system is, within the DMM model, represented by a range of possible sub-patterns, yielding additional information about the speaker’s developmental pathway. The current study identified a large cluster of attachment narratives—more than half the sample—in which the child used one of more of the high-subscript attachment strategies (A3-6), with a significant proportion of the children using or trying to use the A6 (compulsively self-reliant strategy). Patricia Crittenden has described the developmental pathway that leads to the use of the high-subscript A strategies (Crittenden, 1999; Crittenden and Landini, unpublished manuscript^2^). In infancy, the attachment figure is insensitive and predictably unresponsive to the infant’s distress. In response, the infant inhibits displays of negative affect and, instead, looks away from the attachment figure when distressed (avoidant attachment in infancy). In the preschool years, the attachment figure responds protectively if the child is in danger but is not tolerant of negative affect when the child is safe. The child consequently continues to inhibit displays of negative affect when uncomfortable or distressed. However, because avoidance (looking away) is no longer acceptable to the attachment figure—is perceived to be rude—the child learns to please the attachment figure by engaging in preferred activities and by displaying positive affect when engaged in such activities (Type A1-2 strategy). If the A1-2 strategy does not enable the child to obtain sufficient protection and comfort in the attachment relationship—because the parent continues to be predictably displeased with the child or even angry or rejecting—the child may need to utilize a higher-subscript Type A attachment strategy. Children using the A3 strategy (compulsive caregiving) “inhibit negative affect and protect themselves by protecting their attachment figure. In childhood they try to cheer up or care for sad, withdrawn, and vulnerable attachment figures” (p 40) (Crittenden and Landini, unpublished manuscript^2^). Children using the A4 strategy (compulsive compliant) “try to prevent danger, inhibit negative affect and protect themselves by doing what attachment figures want them to do,” especially if the attachment figures are “angry and threatening” (p 40) or if they withdraw love and approval when the child does not comply with expectations. Finally, if the A3-4 strategies fail to elicit comfort and protection, the older school-age child/adolescent may come to use the A6 strategy (compulsively self-reliant). Usually, the A6 “strategy develops in adolescence after [children have] discovered that they cannot regulate the behavior of important but dangerous or non-protective, caregivers” (p 44). Children using the A6 strategy “inhibit negative affect and [seek to] protect themselves by relying on no one other than themselves. This protects the self from dangers posed by reliance on others, but at the cost of loss of assistance and comfort. They withdraw from close relationships as soon as they are old enough to care for themselves” (p 44). In adolescence, because young people using the A6 attachment strategy find close friendships uncomfortable or unnecessary, they avoid such connections and find it difficult to maintain enduring romantic relationships.

This theme of self-reliance or disconnection from attachment relationships—narratives that did not “give importance to attachment experiences” (p 8) (Giovanardi et al., 2018)—was also identified by Giovanardi et al. (2018) in the attachment narratives of adults with gender dysphoria coded using the ABCD method.

The current study identified a second cluster of narratives—from over a quarter of children—that used the C5 strategy (punitively obsessed with revenge). Crittenden has described the developmental pathway that leads to the use of the C5 strategy (Crittenden, 1999; Crittenden and Landini, unpublished manuscript^2^). In infancy, the infant’s attachment figures respond inconsistently and unpredictably to the infant’s signals of distress. The infant remains very distressed and expresses distress at increasingly low thresholds of arousal with great intensity (C/ambivalent infant attachment). In the preschool years, the capacity for disarming (coy) behaviors—which emerge from about 18 months of age—enables the child to use alternating displays of anger, fear, or disarming behaviors to help increase parental predictability and responsiveness (the Type C1-2 strategies). If the C1-2 strategies fail to elicit comfort and protection—that is, if attachment figures continue to be unpredictably responsive to the child’s signals of anger and distress—the preschool or school-age child may increase the intensity of the affective displays. Angry behavior escalates to aggression, and disarming behavior into feigned helplessness, both of which function to coerce attachment figures into responding (the Type C3-4 strategies). Finally, if the C3-4 strategies fail to elicit comfort and protection, the older school-age child/adolescent may use the C5-6 strategies, where negative affect—fear, anger, or desire for comfort—drives the strategy but is no longer signaled in an open and direct way. The C5-6 strategies involve active deception and the distortion of information. Attachment figures are blamed, and the self is exonerated from any blame. In some C5 narratives the child’s anger and derogation involves both parents, and in other narratives it involves anger and derogation of one parent and idealization of the other (triangulated C5). In both cases, information provided in recounted episodes (episodic memory) does not substantiate the speaker’s summation of the situation (semantic memory). In the C5 strategy the expression of anger tends to be intense and yet controlled, with a vengeful quality, whilst in the C6 strategy anger is inhibited, and vulnerability is greatly exaggerated in order to elicit rescue. From adolescence onward, both the C5 and C6 attachment strategies increase the probability of relationship instability, and the C5 strategy, in particular, often leads to involvement in bully victim pairs characterized by repeating cycles of anger and submission.

In the C5 triangulated subgroup, the children were raised in family contexts characterized by high levels of conflict (sometimes escalating to domestic violence) and a pattern in which either a parent (usually the father) had left the family or the child’s contact with a parent had been lost or cut, whether because of marital breakdown, violence, parental mental illness, abuse or suspected sexual abuse of the child, death of the parent, or a parent’s inability to accept the child’s transgender identity. It appears that for the children living is such conflict-ridden families, the triangulated C5 strategy functioned to enable them to give allegiance to (and to idealize) the caregiver with whom they lived (usually the mother)—and on whom they depended for their day-to-day well-being—whilst cutting off (and derogating) the caregiver who had been extruded from the family (usually the father). The intensity of the family conflict—the strong push to take sides—made it difficult for the child to maintain allegiance to both parents because any attempt to hold a more balanced perspective would have put the child at risk of anger and rejection from the caregiver on whom they were dependent.

Along the same lines, in their study of attachment narrative of *adults* with gender dysphoria (coded using the ABCD method), Giovanardi et al. (2018) also identified a cluster of narratives characterized by high levels of anger and derogation toward one attachment figure (often the father) and idealization toward the other attachment figure (often the mother). This suggests that a subgroup of children and adolescents with gender dysphoria will carry forth the use of the triangulated C5 strategy into adulthood.

In the current study, we compared our cohort of children presenting with gender dysphoria not only to children from a non-clinical group but to a cohort of school-age children presenting with mixed psychiatric disorders (the mixed psychiatric group), who had presented to the same hospital department some years before (Crittenden et al., 2010). On patterns of attachment and on rates of unresolved loss and trauma and of the depression modifier, the children with gender dysphoria were indistinguishable from children with mixed psychiatric diagnoses. These findings cohere with those of Giovanardi et al. (2018), who likewise found that attachment narratives of adults with gender dysphoria were indistinguishable from other clinical cohorts.

In the current study we also identified high levels of distress in children presenting with gender dysphoria. Whilst distress pertaining to gender or to bullying or stigmatization in relation to gender is often highlighted in the published literature, linguistic analysis of attachment narratives highlighted that the children also experienced high levels of distress—coded as unresolved loss or trauma and as the depression modifier—pertaining to family conflict, lack of protection by the attachment figure, loss events, maltreatment, domestic violence, and rejection or abandonment by attachment figures. Likewise the children’s distress was palpable in the developmental histories given by children and families because of the high number of ACEs embedded in the story told by the child and family (see Table 2). What these data suggest is that the child’s presentation with gender dysphoria must be considered in the context of the child’s lived experience, the lived experience (via epigenetics) of previous generations, and the complex and non-linear pathways by which such experiences shape health and well-being (Nelson, 2013).

Finally, in the current cohort, alongside the presenting symptom of gender dysphoria, seven-eighths of the children also suffered from one or more comorbid mental health disorders: depression, anxiety, behavioral disorder, and autism. This finding coheres with other studies showing that gender dysphoria is highly comorbid with a broad range of mental health symptoms and disorders (Reisner et al., 2015; Holt et al., 2016; Van Der Miesen et al., 2016; Bechard et al., 2017; Mann et al., 2019; Strauss et al., 2020a; Warrier et al., 2020) and with an emerging literature showing an association between ACEs and multimorbidity—the presence of two or more long-term health (including mental health) conditions (Barnett et al., 2012; Donkin et al., 2018; Hanlon et al., 2020).

The findings from current study highlight some important themes that interweave with the broader literature pertaining to the health and well-being of children in the twenty-first century. First, the increase of children presenting to health services with gender dysphoria is part of a broader pattern, an increase of young people presenting to health services with distress and a range of psychiatric symptoms—anxiety, depression, suicidal ideation, self-harm, suicide attempts, and autism—often comorbid one with another (Weinberger et al., 2018; World Health Organization [WHO], 2019; Maenner et al., 2020; May et al., 2020). Second, because resilience is related to a history of supportive care (Sroufe, 2013), at-risk attachment is a risk factor for developing distress, difficulties with adaptation, and psychiatric disorders generally (Carlson, 1998; Kobak et al., 2006; Crittenden et al., 2010; Spruit et al., 2020)—and also, as is now coming into focus, for gender dysphoria. Third, it has been well documented that ACEs are *inversely* related all of the following: health and well-being across development (Viner et al., 2012; Kivimaki et al., 2020); the family’s socioeconomic status (SES); and the parents’ capacity to nurture and protect their children (Allen et al., 2014; Marmot, 2017). All these factors are, indeed, closely interrelated. Fourth, as we now coming to know through advances in epigenetics, childhood experiences—from the time of conception—are biologically embedded and play an important role in shaping not only the child’s own development but also that of future generations (Dudley et al., 2011; Babenko et al., 2015; Shonkoff, 2016).

Taken together, the above themes highlight that, whist protective attachment relationships and health-promoting family, social, and political environments provide a strong foundation for health and well-being, stressed relationships and unsafe or disrupted family, social, and political environments set the stage for ill health, distress, and mental health disorders, in both current and future generations. From this broader perspective, the child’s presentation with gender dysphoria, along with its comorbidities, should be seen as reflecting both an individual pattern of adaption or maladaptation (to circumstances that vary widely from individual to individual) and a generational pattern of adaption or maladaptation, or what Fausto-Sterling (2012) has called “a pattern in time” (p. 405). This pattern of presentation reflects the coming together of multiple processes—biological, psychological, relational, sociopolitical, and cultural—that have combined to shape the child’s developmental pathway and the pathways of peers at a particular time.

It is also important to see gender dysphoria within a broader sociopolitical and historical perspective. Sir Marmot (2020)—Chair of the Commission on Social Determinants of Health set up by the World Health Organization in 2005—observed that, “So strong is the relation between social determinants and the health of societies that health and health inequalities tell us something fundamental about how well society is meeting the needs of its members” (p 1414) (Marmot, 2020). From this sociopolitical perspective the increase of children presenting to health services with anxiety, depression, autism, obesity, gender dysphoria, and other forms of distress or ill health potentially reflects what Marmot refers to as “fault lines” in contemporary society (Marmot, 2020), the erosion of the good society (Marmot, 2017), and the long-term fallout, or “slow burn” (p 1413) (Marmot, 2020), of unsafe or disrupted family, social, and political environments. In this context, whilst it is important to develop services that support children and adolescents presenting with gender dysphoria—and their families—it is also important to conceptualize the phenomenon of gender dysphoria in a broader relational and sociopolitical context, and to support sociopolitical initiatives that prioritize children’s’ health and well-being more generally (Donkin et al., 2018).

An important limitation of the current study is that careful evaluation of intergenerational risk factors—for example, the quality of the parent’s family environment and the presence of ACEs in the lives of both the parents and grandparents—was not undertaken. Because intergenerational processes and the biological embedding of these processes are likely to be an important theme in the increasing rates of gender dysphoria, mental health comorbidities, and physical health disorders (Dudley et al., 2011; Babenko et al., 2015; Golding et al., 2019), they may be an important focus of future research. Another limitation is the cross-sectional nature of this study, which does not allow us to make causal inferences or to track the point in time (in terms of developmental trajectories) that attachment begins to contribute to the shaping of the child’s development—and brain development, in particular. Large, prospective studies—with multiple points of data collection, running from birth to adulthood—are better suited to untangling the contributions from multiple interacting factors. Finally, our clinician-based ratings of distress were done by clinician consensus and did include formal inter-coder reliability. Despite these limitations, the study also has significant strengths. Most notably, through a rigorous and respected methodology relying not of self-reports but on structured interviews and DMM linguistic analysis, we were able to identify the attachment patters of a sizable series of children who presented for assessment of gender dysphoria to the psychological medicine component of our multidisciplinary gender service.

In summary, the findings from the current study suggest that gender dysphoria in children arises in association with developmental pathways—reflected in at-risk patterns of attachment and high rates of unresolved loss and trauma—that are shaped by disruptions to family stability and cohesion, ACEs (including maltreatment), and SES (Golden and Oransky, 2019; Meyer-Bahlburg, 2019; Alonso-Zaldivar, 2020). Alongside other studies and perspectives (Bechard et al., 2017; Giovanardi et al., 2018; Churcher Clarke and Spiliadis, 2019; de Graaf and Carmichael, 2019; D’Angelo, 2020), this study confirms the importance of conceptualizing gender dysphoria by using a broad lens that takes into account the multiple factors that contribute to the child’s distress, difficulties with adaptation, multimorbidity, and loss of health and well-being. From this broader perspective, neurobiological explanatory models of gender dysphoria must account for the child’s lived experience—and that of preceding generations—in shaping brain development and in shaping brain networks involved in “own-body and self” (Altinay and Anand, 2020) experiences. Likewise, treatment interventions with these children require a comprehensive biopsychosocial assessment with the child and the family, followed by therapeutic interventions that address, insofar as possible, the breadth of factors that are interconnected with each particular child’s clinical presentation. Included in this context are efforts to increase the child’s sense of acceptance by, and safety with, family and peers.

## Data Availability Statement

The datasets presented in this article are not readily available because ethics to place data in a public repository was not obtained from the children and families who participated in this study. The data may potentially be made available on request to the authors after review of the request by the Children’s Hospital Network Ethics Committee. Requesters should submit a data analysis plan before requesting the data. Requests to access the datasets should be directed to KK, kasia.kozlowska@health.nsw.gov.au.

## Ethics Statement

The studies involving human participants were reviewed and approved by the Sydney Children’s Hospital Network Ethics Committee. Written informed consent to participate in this study was provided by the participants’ legal guardian/next of kin.

## Author Contributions

KK: conception of the work, psychological data collection, data analysis and interpretation, drafting the article, and teaching to fund raise. CC: conception of the work, psychological data collection, contributions to draft revisions, and teaching to fund raise. GM: psychological data collection, contributions to draft revisions, and teaching to fund raise. AM: medical data collection, contributions to draft revisions, and teaching to fund raise. GA: medical data collection, contributions to draft revisions, and teaching to fund raise. All authors contributed to the article and approved the submitted version.

## Conflict of Interest

The authors declare that the research was conducted in the absence of any commercial or financial relationships that could be construed as a potential conflict of interest.

## Footnotes

1 Birkenfeld-Adams’s study, published as a dissertation, is reported in Goldberg (1997). “Attachment and childhood behavior problems in normal, at risk and clinical samples,” in Attachment and Psychopathology, eds. A. Leslie and K. J. Zucker (New York: Guilford Press), 171–195.

2 Crittenden, P.M. and Landini, A. (2001–2006). School-aged assessment of attachment. Coding manual using the dynamic maturational method. (© Crittenden, P. M. and Landini, A. Available from the author).
